# *Toxocara canis* infection in multiple types of animals: ophthalmological and pathological observations

**DOI:** 10.1186/s13071-023-06070-y

**Published:** 2024-02-23

**Authors:** Shuxin Zheng, Limei Sun, Li Huang, Yue Xie, Xiaoyan Ding

**Affiliations:** 1 State Key Laboratory of Ophthalmology, Zhongshan Ophthalmic Center, Sun Yat-Sen University, 7 Jinsui Road, Guangzhou, 510060 China; 2 Guangdong Provincial Key Laboratory of Ophthalmology and Visual Science, 7 Jinsui Road, Guangzhou, 510060 China; 3 Department of Parasitology, College of Veterinary Medicine, Sichuan Agricultural University, 211 Huimin Road, Chengdu, 611130 China

**Keywords:** Ocular toxocariasis, *Toxocara canis*, Ophthalmology, Pathology

## Abstract

Human ocular toxocariasis (OT), caused by pet roundworm *Toxocara canis* (Nematoda Ascaridoidea), is a worldwide ocular parasitic infection that poses a severe threat to eyesight, especially in school-aged children. However, the infection process and pathological mechanism of *Toxocara* are difficult to study in the human body. This study was designed to explore long-term ocular manifestations in different rodents infected with *Toxocara canis*, uncovering the specific pathological mechanism and migration pathway of larvae after infection. The three types of experimental animals we selected were C57BL/6 mice, Mongolian gerbils and Brown Norway rats. Mice were randomly divided into five groups and infected orally with 1000, 2000, 4000, 8000 and 10,000 *T. canis* eggs; gerbils were randomly divided into four groups and infected orally with 1000, 2000, 4000 and 10,000 *T. canis* eggs; rats were randomly divided into three groups and infected orally with 2000, 6000 and 10,000 *T. canis* eggs. Their ocular changes were closely observed and recorded for at least 2 months. We also enucleated the eyeballs of some animals to perform pathological sectioning and hematoxylin-eosin staining. After 3 dpi (days post-infection), hemorrhagic lesions, mechanical injury of the retina and larval migration could be observed in some infected animals. The ocular infection and mortality rates tended to be stable at 7 dpi. Larval tissue, structure disorder and inflammation could be observed in the pathological sections. In conclusion, the mice infected with 2000 *T. canis* eggs and gerbils infected with 1000, 2000 and 4000 *T. canis* eggs showing obvious ocular lesions and lower mortality rates could provide a basis for long-term observation.

**Graphical Abstract:**

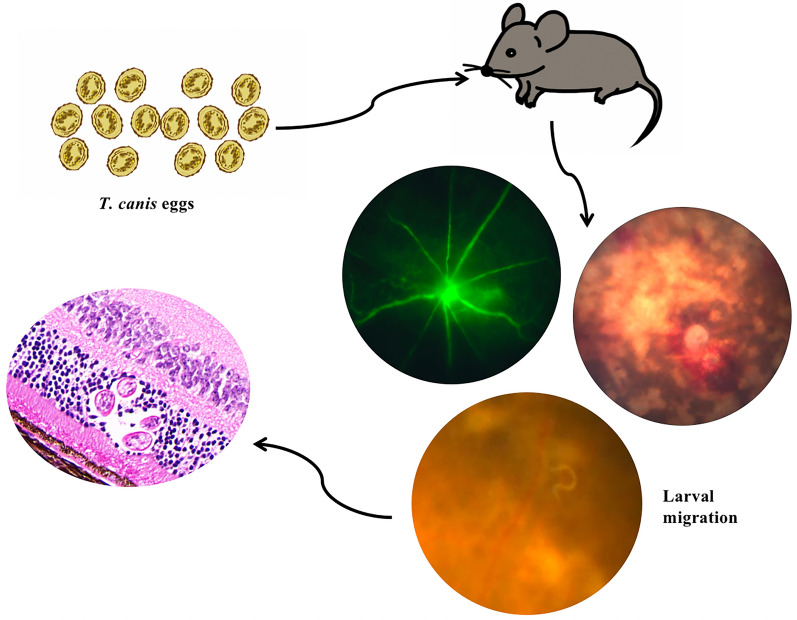

**Supplementary Information:**

The online version contains supplementary material available at 10.1186/s13071-023-06070-y.

## Background

The infection or migration of parasites to the eye is a major cause of blindness worldwide [1]. Toxocariasis is one of the most common zoonotic infections mostly caused by *Toxocara canis* and less frequently by *Toxocara cati* [2], and the definitive hosts are dogs and cats. Paratenic hosts include humans, mice, rats, lambs, poultry, pigs, flies and cockroaches [3]. Ocular toxocariasis (OT) is the clinical manifestation of ocular infection caused by *Toxocara* larvae. According to Badri’s report [4], the pooled prevalence of OT in humans is 9%. In immunological studies, the highest incidence is in Africa and the lowest in the Mediterranean region. Patients with OT are usually affected unilaterally, although occasionally cases of bilateral disease have been reported. The most common complaint is blurry vision or loss of vision [5]. OT is a common cause of vision loss, especially for school-aged children [6]. People in developing countries and places with poor sanitation, as well as people who own dogs or cats as pets, are most likely to be infected [7].

The toxocariasis transmission cycle begins when an infected dog or cat sheds unembryonated eggs in their feces. Eggs are not infectious until embryonation 2–4 weeks later [8]. Humans can become infected by ingesting infectious eggs, which are found in soil contaminated by dog and cat feces. Also, undercooked meat of infected animals can cause infection [9, 10]. Ingestion results in hatching of the eggs, and the larvae penetrate the intestinal wall before being carried by the circulation to a variety of tissues, including liver, heart, lungs, brain, muscle and eyes [10, 11]. Toxocariasis can be asymptomatic or result in severe organ damage. Clinically evident human toxocariasis comprises three essential clinical types: visceral larva migrans (VLM), neurological larva migrans and ocular larva migrans (OLM; also known as OT) [12, 13]. The most common clinical forms are VLM and OT [14]. Patients with VLM can present with fever, hepatomegaly, splenomegaly, respiratory symptoms, muscular pain and anorexia associated to eosinophilia and high titers of anti-*Toxocara* antibodies [8].

At present, the larval migration and specific pathological mechanism of OT are still not clear. Previous animal models of OT include mice [15, 16], rats [17, 18], rabbits [19], gerbils [18, 20, 21], hamsters [22], guinea pigs [23] and monkeys [24]. However, these studies revealed low incidence of ocular infection and lacked systematic observation and comparison [20], and the period of most experimental studies was short. In this study, we carried out oral inoculation of *T. canis* in C57BL/6 mice, Mongolian gerbils and Brown Norway (BN) rats, observed their eye manifestations and conducted fundus angiography and pathological change exploration, aiming to observe long-term eye changes in multiple types of animals infected with *T. canis*.

## Methods

### Animals

Male C57BL/6 mice aged 3 weeks and male BN rats aged 1 month were purchased from SPF Biotechnology (Beijing, China); male gerbils aged 2 months were purchased from Hangzhou Medical College (Hangzhou, China). The animals were housed under specific pathogen-free and standard lighting conditions (12 h alternating day and night cycles) at the Sichuan Agricultural University Veterinary Medical Laboratory, and they had free access to food and water. The animals were maintained and infected in accordance with institutional and national guidelines. This study was approved by the Animal Ethics Committee of Sichuan Agricultural University (China; approval no. SYXK 2014–187).

### Parasites

The infective *T. canis* eggs were provided by the parasite laboratory of Sichuan Agricultural University. In brief, the male and female adult *T. canis* worms were obtained from the intestines of stray dogs without any deworming treatment under the Scientific Procedures Premises License for the College of Veterinary Medicine, Sichuan Agricultural University. Then, these adult worms were cultured in RPMI1640 media containing 200 µg/ml penicillin, 200 units/ml streptomycin and 10 µg/ml amphotericin B at 37 °C overnight for mating. The fertilized eggs were harvested, followed by dilution using saline containing 200 µg/ml penicillin, 200 units/ml streptomycin and 10 µg/ml amphotericin B. Eggs (10 µg/ml) were placed in a 35-mm-diameter culture plate under at 28° C for 21 days. Egg developments into the third larval stage were observed with optical microscope. Infective eggs were harvested, counted and resuspended in phosphate-buffered saline (PBS) with different concentrations for use.

### Experimental grouping and infection

We referred to previous reports on the infection dose of animals [15, 18, 20, 21]. C57BL/6 mice were divided randomly into five groups (M1k, M2k, M4k, M8k, M10k) and orally infected with 1000, 2000, 4000, 8000 and 10,000 *T. canis* eggs in a total volume of 0.1 ml PBS, respectively. Gerbils were divided randomly into four groups (G1k, G2k, G4k, G10k) and orally infected with 1000, 2000, 4000 and 10,000 *T. canis* eggs in a total volume of 0.1 ml PBS, respectively. BN rats were divided randomly into three groups (R2k, R6k, R10k) and orally infected with 2000, 6000 and 10,000 *T. canis* eggs in a total volume of 0.1 ml PBS, respectively.

### Ophthalmological examination

Ophthalmological examination of all infected animals was carried out with pupils dilated with tropicamide (Santen Pharmaceutical, Japan) and corneas protected by Gatifloxacin Ophthalmic Gel (Shenyang Xingqi eye medicine Co., Ltd) under anesthesia with sodium pentobarbitone (50 mg/kg, Shanghai Hailing Biotechnology Co., Ltd). Comprehensive ocular examinations were performed as follows: the anterior segment was examined by portable slit lamp, and the fundus was examined by MICRON IV retinal microscope (Phoenix Micron IV, Phoenix Research Labs, CA, USA); retinal vascular system was examined using MICRON IV retinal microscope after intraperitoneally injection with 2% fluorescein sodium (Health Manufacturing Services B.V., USA). After ocular observations, the animals were placed on the heat preservation pad for resuscitation, and then tobramycin eye ointment was applied to both eyes. The animals were put into cages after they were fully awake. All animals were observed and recorded, and the observation interval was every 2 h within 3 dpi, every 2 days between 4 and 14 dpi and every 1 week after 14 dpi. According to previous literature, the migration of *Toxocara* larvae in paratenic hosts can be divided into three phases, namely the acute, subacute and chronic phases [25]. The observation period was 4 months for C57BL/6 mice and 2 months for gerbils and rats.

### Histopathological examination

Eyeballs were enucleated immediately after cervical dislocation under anesthesia and fixed in FAS eyeball fixative solution or paraformaldehyde for more than 24 h. Serial section was taken and stained with hematoxylin and eosin.

### Statistical analysis

Analysis was performed using IBM SPSS Statistics version 25.0 (IBM Corporation, Armonk, NY, US). The data were presented as frequency and percent (%).

## Results

### Incidence of ocular infection

**Fig. 1:**
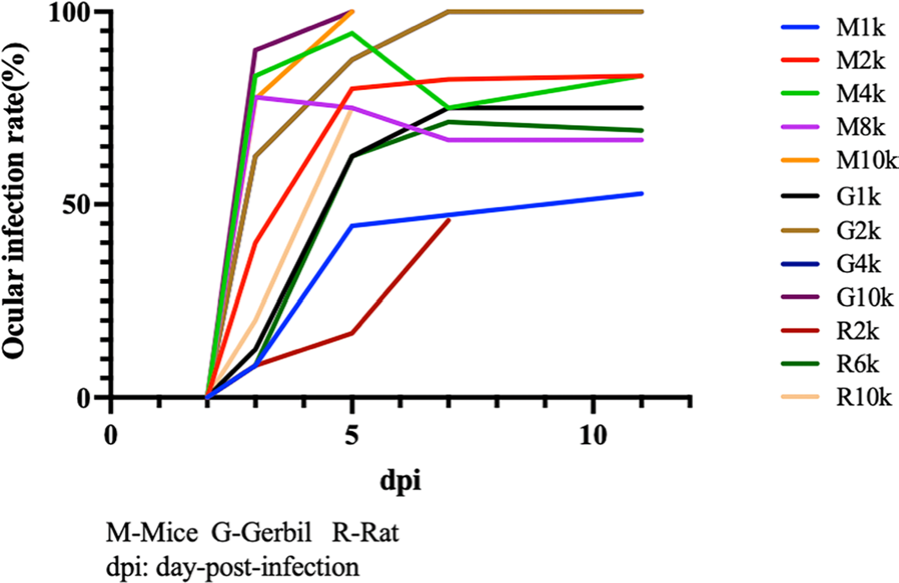
Ocular infection rate

The ocular infection rates at 3, 5, 7, 14, 35 and 56 days after *T. canis* challenge are shown in Table 1. No ocular lesions were found in animals before 3 dpi. Afterwards, in mice, the ocular infection rate in M1k and M2k increased steadily after infection although many infected mice died in M4k and M8k within 5 dpi. In gerbils and BN rats, the ocular infection rates reached a high level and stabilized at 5–7 dpi. The ocular infection rates of G1k, G2k, G4k, R2k and R6k groups seemed to increase during this infection course. The four groups M4k, M8k, M10k and G10k had the highest ocular infection rates of, 83.3, 77.8, 77.3 and 90%, respectively, at 3 dpi (Table 1 and Fig. 1). The animals in M10k, G10k and R10k were all dead within 5 dpi because of systemic parasitic infection. The mortality varied by group within 7 dpi and the animal deaths usually occurred within 7 dpi, then the mortality tended to be stable. It was clear that a higher infection dose led to a higher death rate.

**Table 1:** Ocular infection rate of all groups at each time point

Group	2 dpi (%)	3 dpi (%)	5 dpi (%)	7 dpi (%)	14 dpi (%)	35 dpi (%)	56 dpi (%)
M1k	0.0	8.3	44.4	47.2	55.6	45.8	54.5
M2k	0.0	40.0	80.0	82.4	75.0	95.5	100.0
M4k	0.0	83.3	94.4	75.0	66.7	66.7	100.0
M8k	0.0	77.8	75.0	66.7	75.0	66.7	100.0
M10k	0.0	77.3	100.0	–	–	–	–
G1k	0.0	12.5	62.5	75.0	37.5	62.5	66.7
G2k	0.0	62.5	87.5	100.0	100.0	100.0	–
G4k	0.0	62.5	87.5	100.0	100.0	100.0	100.0
G10k	0.0	90.0	100.0	–	–	–	–
R2k	0.0	8.3	16.7	45.8	–	–	–
R6k	0.0	8.3	62.5	71.4	69.2	56.3	50.0
R10k	0.0	20.0	75.0	–	–	–	–

*M* mouse, *G* gerbil, *R* rat, *dpi* days post-infection

### Ocular lesions

**Fig. 2:**
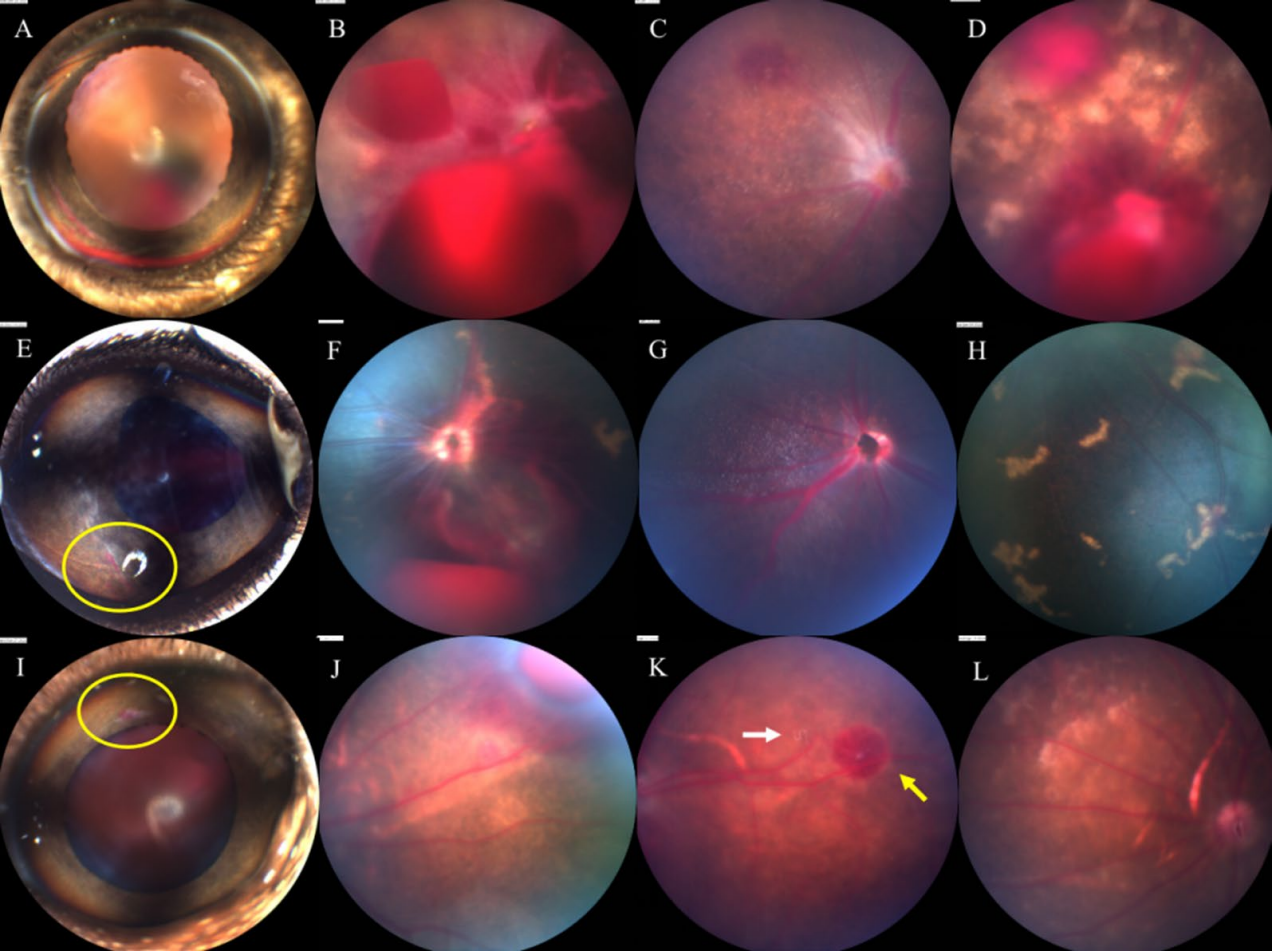
Ocular lesions. **A** Anterior segment of a C57 mouse at 5 dpi shows hyphema. **B** Fundus of a C57 mouse at 5 dpi shows preretinal hemorrhage and retinal hemorrhage. **C** Fundus of a C57 mouse at 11 dpi shows vitreous opacity and retinal hemorrhage. **D** Fundus of a C57 mouse at 5 dpi shows hemorrhagic lesions and retinal lesions. **E** Anterior segment of a gerbil at 3 dpi shows pupil deformation and slight iris bleeding. **F** Fundus of a gerbil at 5 dpi shows preretinal hemorrhage and retinal hemorrhage. **G** Fundus of a gerbil at 11 dpi shows vitreous opacity. **H** Fundus of a gerbil at 5 dpi shows retinal lesions. **I** Anterior segment of a BN rat at 3 dpi shows slight iris bleeding. **J** Fundus of a BN rat at 10 dpi shows preretinal hemorrhage. **K** Fundus of a BN rat at 8 dpi shows retinal hemorrhage (yellow arrow); larvae could also be found in retinal layer (white arrow). **L** Fundus of a BN rat at 5 dpi shows retinal lesions

**Fig. 3:**
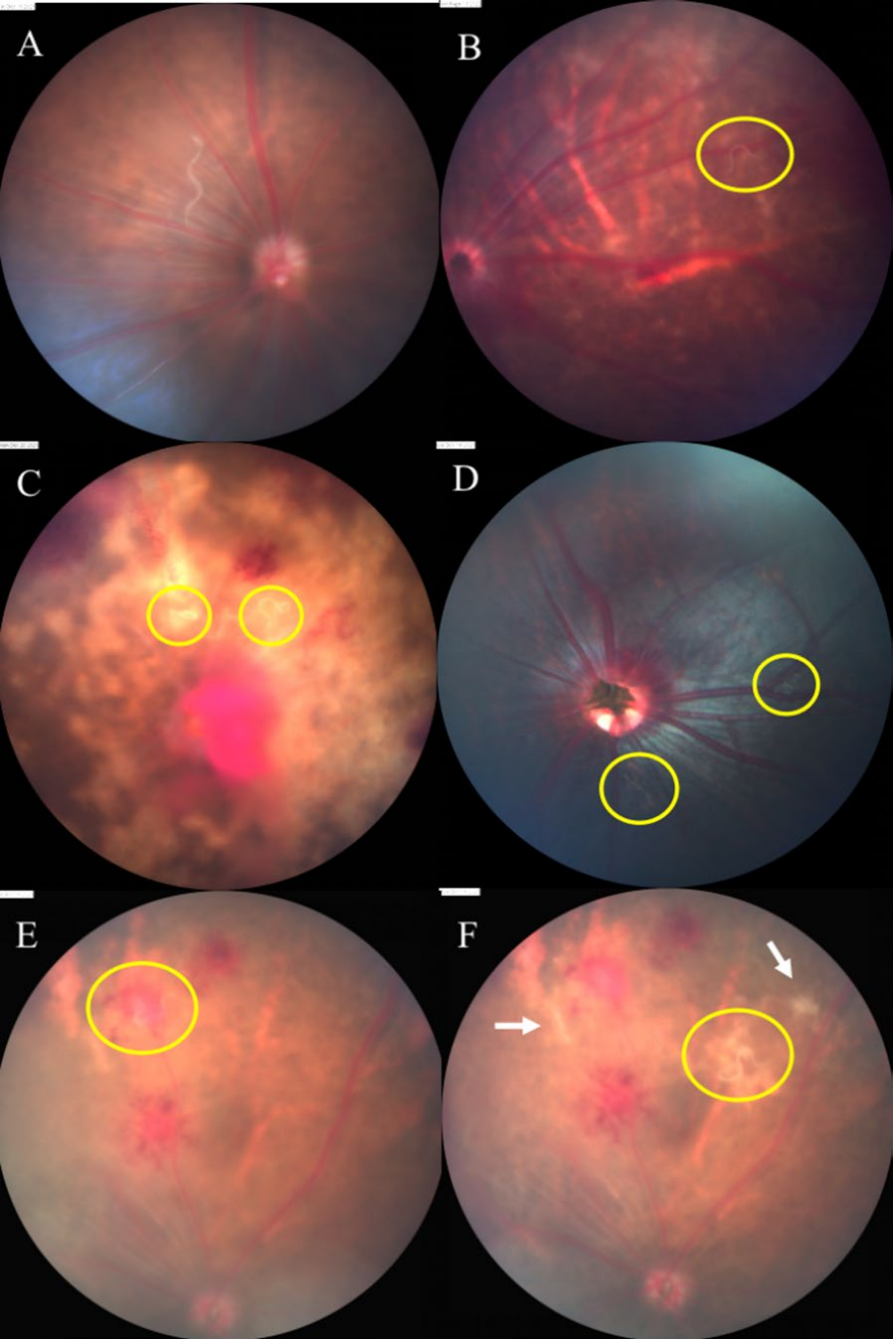
Larval migration. **A** Fundus of a C57 mouse at 3 dpi shows a larva moving in vitreous. **B** Fundus of a BN rat at 5 dpi shows a larva moved in vitreous. **C** Fundus of a C57 mouse at 5 dpi shows vitreous opacity, hemorrhagic lesions and retinal lesions. Two larvae could be observed wriggling between the retinal layers. **D** Fundus of a gerbil at 3 dpi shows optic disc hemorrhage and two larvae moving between the layers of the retina. **E**, **F** Trace left by larvae in fundus of C57 mice at 3 dpi (white arrow)

In our study, hyphema, preretinal hemorrhage, retinal hemorrhage, retinal lesions, vitreous opacity and larva migration in vitreous or subretinal tissues were observed across the whole infection process (Figs. 2, 3). The abnormality of retinal vessels was detected by fluorescein angiography after 3 dpi. Among the ocular lesions, hemorrhagic injuries and retinal lesions were the main manifestations in mice, gerbils and rats infected with *T. canis*.

#### Acute phase

The incidence of major ocular manifestations in different 7 dpi groups is shown in Table 2. Hyphema was commonly found in C57 mice (Fig. 2A) but rarely observed in gerbils and rats. Nevertheless, retinal hemorrhage, preretinal hemorrhage, retinal lesions and larval migration could be detected in these three types of animals. Notably, ocular lesions were milder in rats than in gerbils and mice.

**Table 2:** Ocular lesions at 7 dpi

Group	Hyphema (%)	Preretinal hemorrhage (%)	Retinal hemorrhage (%)	Retinal lesion (%)	Vitreous opacity (%)
M1k	0.0	13.9	41.7	38.9	0.0
M2k	14.7	50.0	64.7	70.6	2.9
M4k	25.0	25.0	62.5	62.5	0.0
M8k	0.0	16.7	16.7	33.3	0.0
M10k	–	–	–	–	–
G1k	0.0	12.5	25.0	75.0	0.0
G2k	0.0	12.5	75.0	75.0	0.0
G4k	0.0	25.0	62.5	100.0	0.0
G10k	–	–	–	–	–
R2k	12.5	4.2	41.7	12.5	0.0
R6k	3.6	3.6	64.3	25.0	0.0
R10k	–	–	–	–	–

*M* mouse, *G* gerbil, *R* rat, *dpi* days post-infection

Larval migration was observed in the vitreous (Fig. 3A, B) or retinal layer (Figs. 2K, 3C–F), with the latter predominating. Video of larval migration is shown in Additional file 1. More than one larva was found in the eyes of some mice and gerbils (Fig. 3C, D). However, most larvae eventually moved to the peripheral retina, making it impossible to make continuous observations. The larvae were easily detectable in the gerbils because of their dark grey fundi. Moreover, the larvae that migrated to the vitreous cavity were faster and more active than those in the retinal layer. The larvae can migrate into the retinal layer freely and leave traces on the retina, causing mechanical damage (Fig. 3E, F). Therefore, although we sometimes cannot capture the migration of larvae, we can infer that there have been larvae migrating in the eye based on the traces left by the larvae on the fundus.

#### Subacute and chronic phase

**Fig. 4:**
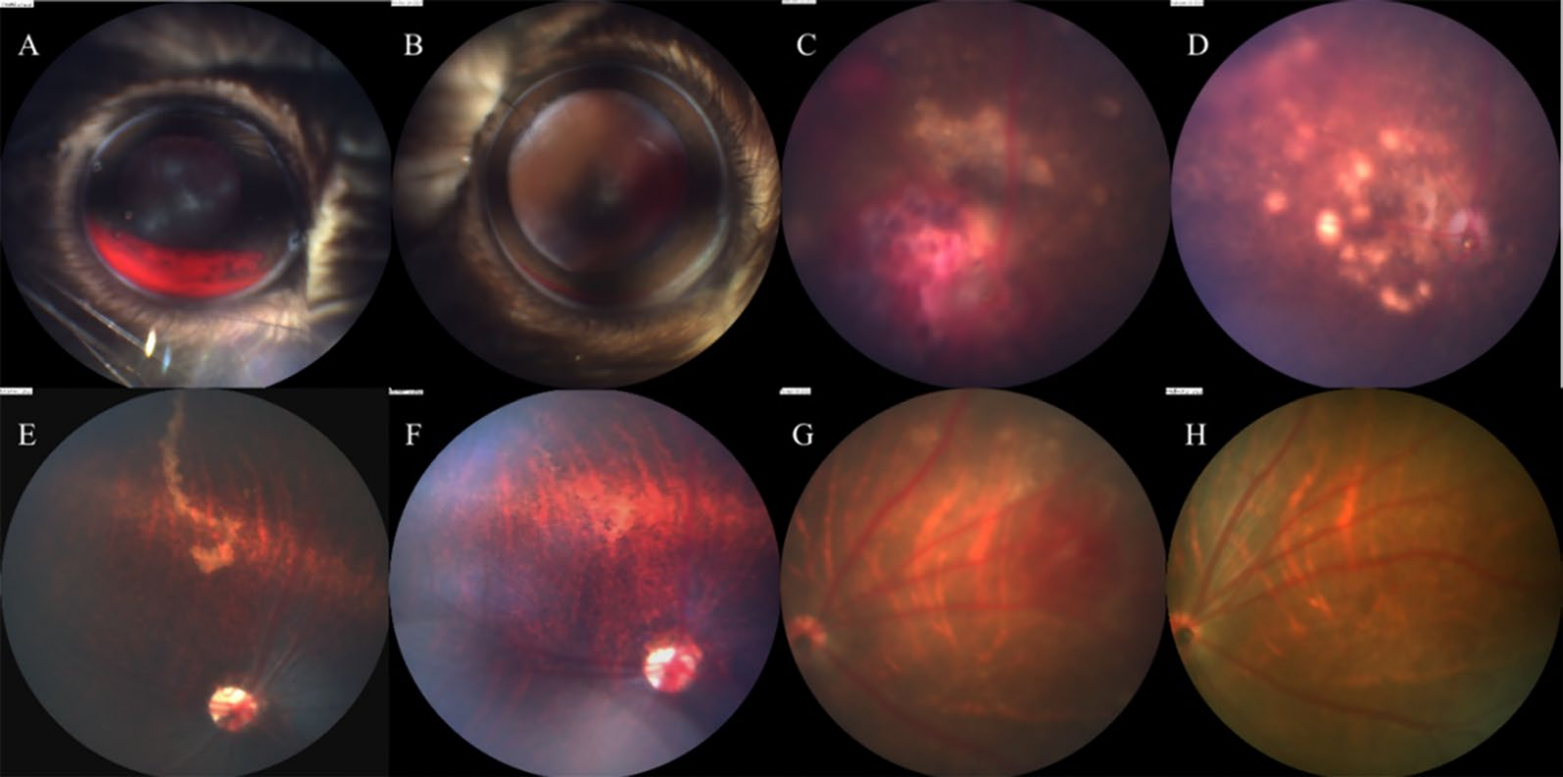
Ocular lesions on mice, gerbils and BN rats from 3 to 65 dpi. **A** Hyphema of a C57 mouse at 3 dpi. **B** The same mouse, anterior segment at 7 dpi; most of hematocele was absorbed. **C** Fundus of a C57 mouse at 5 dpi shows retinal hemorrhage and lesions. **D** The same C57 mouse at 39 dpi; the retinal hemorrhage was absorbed, and residual retinal lesions were left. **E** Fundus of a gerbil at 5 dpi shows yellowish-white lesion of the retina. **F** The same gerbil at 65 dpi; previous retinal damage has almost completely disappeared. **G** Fundus of a rat at 7 dpi shows retinal hemorrhage and slight retinal lesion. **H** The same rat at 28 dpi; the hemorrhagic lesions have been completely absorbed, and retinal lesions have disappeared

As the experiments continue, hyphema, retinal hemorrhage and preretinal hemorrhage were partly or totally absorbed, and retinal lesions were also gradually repaired (Fig. 4). However, vitreous opacification and retinal lesions were observed; retinal detachment occurred in a few animals.

### Fluorescein angiography

**Fig. 5:**
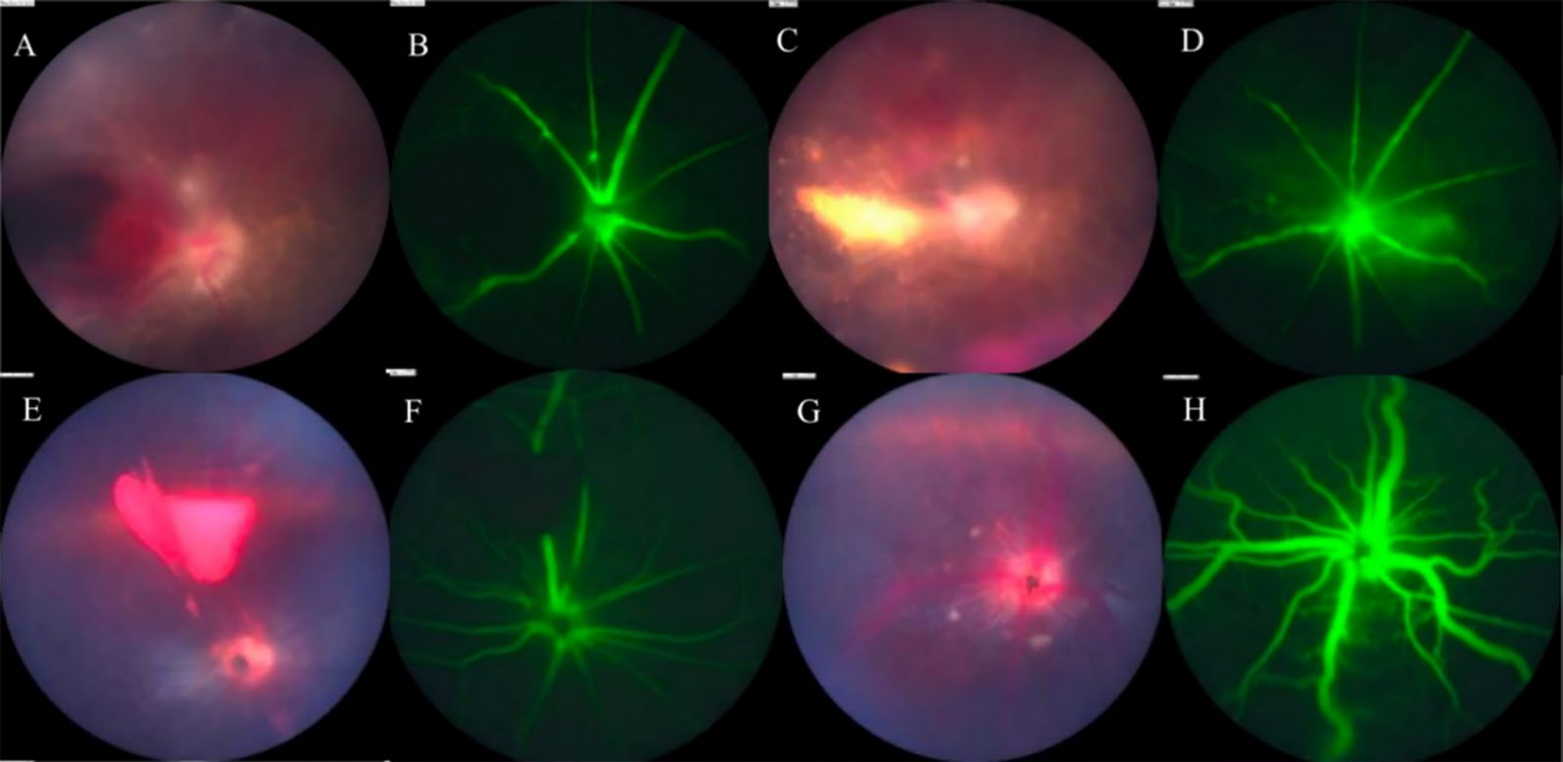
FFA of mice and gerbils. **A**, **B** Fundus of a mouse at 14 dpi, vitreous opacification, preretinal hemorrhage in fundus photography; FFA shows hemorrhage obscured fluorescence, optic disc hyperfluorescence, retinal vascular dilatation and capillary leakage. **C**, **D** Fundus of the same mouse at 28 dpi shows aggravated vitreous opacification; preretinal hemorrhage was absorbed organically; hemorrhage obscured fluorescence and perioptic capillary leakage. **E**, **F** Fundus of a gerbil’s right eye at 28 dpi, preretinal hemorrhage, peripapillary proliferation, vascular white sheaths on the nasal side, and hemorrhage obscured fluorescence. **G**, **H** Fundus of the same gerbil’s left eye, peripapillary proliferation, retinal hemorrhage, vitreous opacity, vascular white sheaths and capillary leakage in FFA

**Fig. 6:**
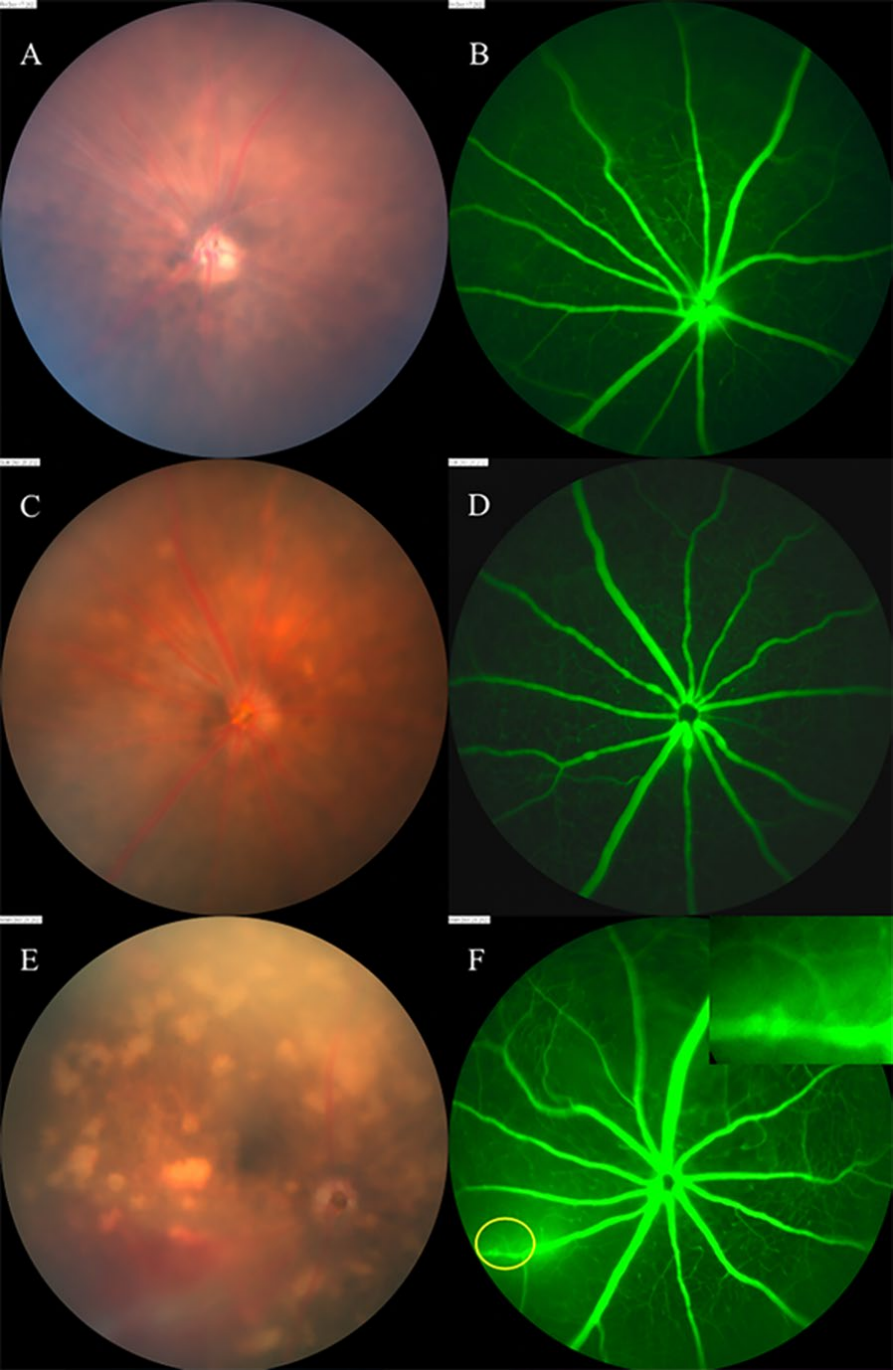
Abnormal changes in blood vessels displayed by FFA. **A** **B** Fundus of a C57 mouse at 2 dpi shows no significant damage, but FFA manifests mild segmental dilation of blood vessels. **C**, **D** Fundus of a C57 mouse at 11 dpi shows no significant damage, but FFA indicates obvious tumor-like dilation of blood vessels near the optic disc. **E**, **F** Fundus of a C57 mouse at 5 dpi shows retinal hemorrhage and lesions; FFA shows vascular rupture

In the fundus of mice and gerbils, hyperfluorescence of the optic disc, vascular leakage, vascular breakage or rupture, vascular tortuosity and dilation, and retinal telangiectasia were detected by fundus fluorescein angiography (FFA) (Figs. 5, 6). FFA could monitor abnormal segmental dilation of blood vessels at 2 dpi before presenting significant eye manifestations (Fig. 6B). The vascular abnormalities in FFA were most severe in mice, followed by gerbils and rats.

### Pathological features

**Fig. 7:**
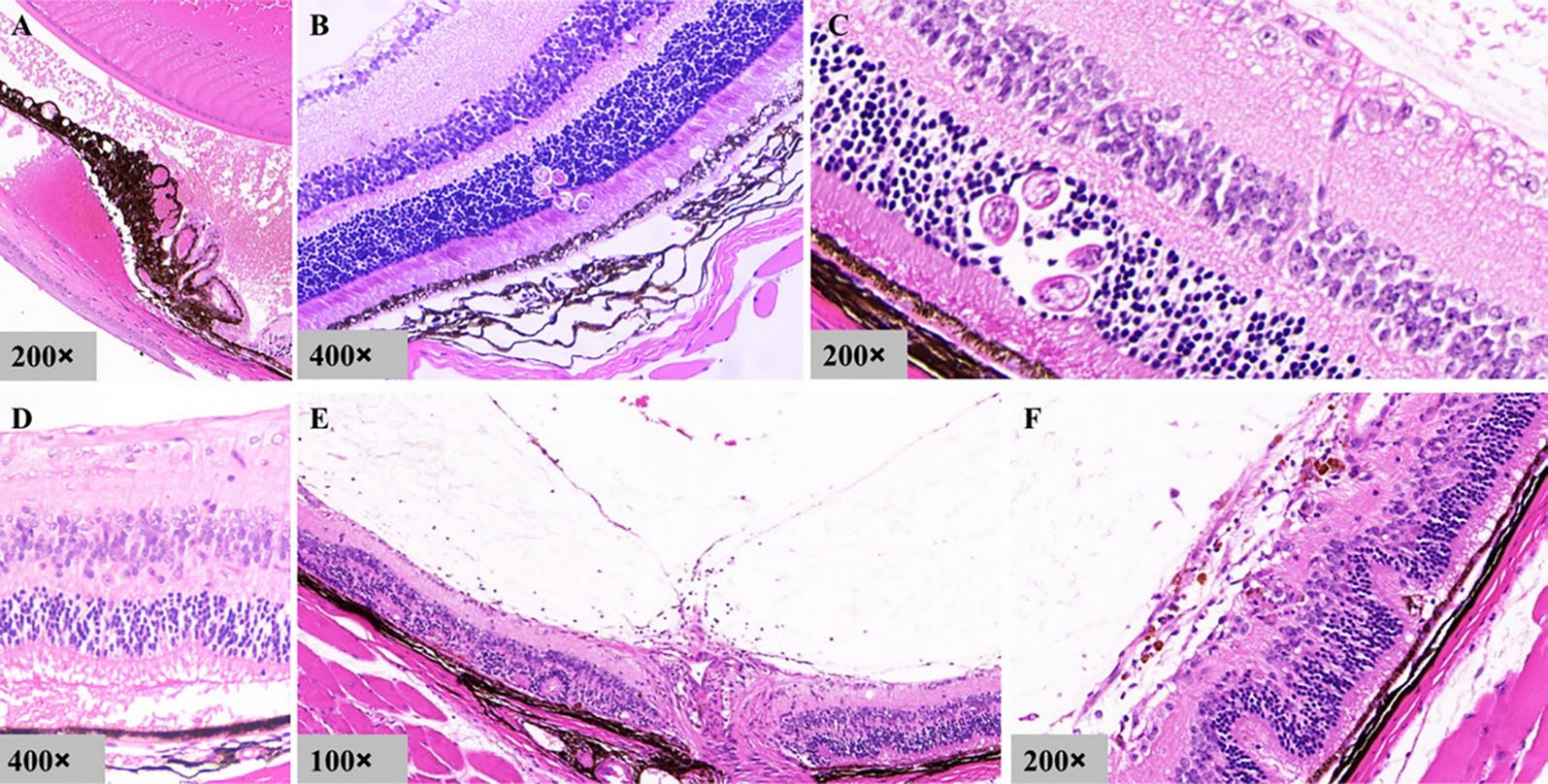
Pathological sections of eyeballs. **A** Hyphema of a C57 mouse at 5 dpi. Dilatation of blood vessels in iris and ciliary body; blood cells accumulate in the anterior chamber. **B**, **C** Larvae located in the outer nuclear layer and photoreceptor cell layer of the retina. **D** A gerbil at 15 dpi. Edema of outer layer of retina. **E**, **F** A C57 mouse at 26 dpi. Derangement and edema of outer layer of retina. Many inflammatory cells infiltrated the optic nerve, anterior optic disc and anterior retina

To identify the retinal layers into which the larvae entered, retinal pathological changes were observed. The results showed that the larvae were mainly located in the outer nuclear layer and the photoreceptor cell layer of the retina (Fig. 7B, C). The outer retina showed vacuolar changes, and the inner retina was thickened with edema (Fig. 7B, D). Encouragingly, in a mouse with hyphema, ciliary vessel dilatation was observed (Fig. 7A). In some animals, the outer layer of the retina was disorganized and edematous, and many inflammatory cells infiltrated the optic nerve, anterior optic disc and preretinal tissue (Fig. 7E, F).

## Discussion

This study presents a comparison of three species infected with *T. canis* for the first time to our knowledge, with long-term comprehensive ophthalmological observation, retinal fluorescein angiography and pathological observation. We also saw active larvae and subsequent mechanical damage of intraocular structures under retinal microscope in all three species throughout the experiment. In summary, animals in the M1k, G1k and G4k groups had higher rates of eye infections and lower mortality rates; this could serve as a basis for long-term observation.

### Infection differences among three animal species

There are three phases which can be used to distinguish the somatic migration of *Toxocara* larvae in paratenic hosts. The acute phase of infection corresponds to the hepato-pulmonary phase, which lasts from the time of infection to approximately 14 dpi. Then, during the subacute phase, which corresponds to the myotropic-neurotropic phase, larvae migrate and accumulate in somatic tissues. Finally, the chronic stage begins at approximately 28 dpi. The larvae migrate throughout the entire body to predilection sites where they can remain infective for months to years [25].

The ocular manifestations appeared at 3 dpi in all tested animals, with more significant changes at 5–7 dpi, which is consistent with previous studies in which the larvae were found to reach the brain as early as 3–5 dpi [26]. From the acute phase, all three species of animals could exhibit various ocular manifestations. Larval migration could be observed in the retina or vitreous throughout the experimental process in these three animal species with more larval activities in the vitreous. After the animal’s death, the peristalsis of the larvae gradually slowed down within a few minutes and finally stopped. We found, for the first time to our knowledge, that the larvae can wriggle in eyes for a long time in living mice, rats and gerbils.

Ocular manifestations varied among the three species. Hyphema, preretinal hemorrhage, retinal hemorrhage, retinal lesion and vitreous opacity were the main manifestations. In mice, hyphema was the most frequent manifestation observed. The lesions were more serious in the acute phase, possibly because of the diameter of the blood vessel through which the larvae migrate. In general, the diameter of the blood vessel in gerbils and rats is larger than that in mice, which reduces the possibility of mechanical damage to the blood vessel wall by the larvae. Additionally, the body size of mice is smaller than that of gerbils or rats; therefore, under the same dosage of infectious eggs, the tolerance of the mouse was worse and the damage more serious.

In our preliminary experiment, we found that at the same infection dose, the ocular manifestations in rats were less severe compared to gerbils and mice. Therefore, we set groups of higher infection dose for rats; however, despite this, the ocular manifestations in rats remained relatively mild. Gerbils are more susceptible to *T. canis*, and their ocular fundus is gray, which facilitates the observation of lesions. Of course, we also found some problems when using gerbils. For instance, gerbils are more easily provoked and difficult to grasp, and more tail breakage occurred during the experiment; moreover, when we took fundus photography, the eyeball of gerbils often rotated, even under anesthesia, which was not conducive to ocular image acquisition. In rats, the ocular fundus was much closer to that of humans, but their ocular lesions were slight. The mortality rate of *T. canis*-infected animals rose with increasing egg doses. Most of animals died with symptoms of shortness of breath and epistaxis; anatomy revealed bleeding in the lungs. The main cause of death was considered to be larval lung invasion. A tremendous dose of larval infection could cause larval load to exceed the immune ability of the body or the limiting ability of the liver; then, larva escaped into the eyes or other organs, causing damages [11].

### Pathological findings

In pathological sections, we observed larval tissues, hemorrhagic changes, ocular vascular dilation and disorganized retinal architecture. The structural disorder and folds of the retina indicated the occurrence of related inflammatory reactions. As the observation time was prolonged, inflammatory cell infiltration was observed, which was consistent with the absorption of hemorrhagic lesions, vitreous opacity and white sheath-like changes in blood vessels during subacute and chronic phases. In the whole observation period, no obvious granuloma lesions were seen in the retina and choroid of mice, gerbils and BN rats.

### Route of larval migration

Possible routes of the larvae entering the eyes were reported in previous studies and included via the arteries, brain to optic nerve or brain to cerebrospinal fluid and then arriving in the choroid [11]. Although there was no direct evidence on larvae passing through the blood vessels in this study, we found a large amount of blood vessel damage in the fundus which resulted in hemorrhagic lesions. Fluorescein angiography showed that some animals could have abnormal segmental dilation (Fig. 6B) or segmental tumor-like dilation of blood vessels (Fig. 6D) before obvious eye damage, and these vascular abnormalities were highly suggestive of vascular inflammation. In addition, the angiography presented alterations suggestive of vascular rupture (Fig. 6F), which may be the mechanical damage caused by larvae. In pathological sections, we also found vasodilatation of the ciliary body (Fig. 7A). Based on the above findings, we speculated that the larvae may enter the eye through the arteries. We guessed that the tortuosity and dilation of the retinal blood vessels may result from an inflammatory reaction caused by proteins secreted or excreted by larvae, which would facilitate their migrations in the blood vessels.

## Conclusions

Hyphema, hemorrhagic lesions, retinal lesions, vitreous opacity, abnormality of retinal vessels and larval migration could be observed across the whole infection process in mice, gerbils and rats infected with *T. canis*. Through comparisons of these animals, we considered that the gerbils infected with 1000, 2000 and 4000 *T. canis* eggs and mice infected with 2000 *T. canis* eggs were the ideal animals for further exploration related to ocular toxocariasis.

### Supplementary Information

**Additional file 1**: Video of larval migration in eyes of animals. In the fundus recording of C57 mouse, two larvae located in the vitreous cavity and retinal layer can be observed by adjusting the focal length. In the fundus recording of gerbil and rat, two larvae can be seen moving in the retinal layer.

## Data Availability

All relevant data during this study are included in the article.
